# Pollen Quantitative and Genetic Competitiveness of Rice (*Oryza sativa* L.) and Their Effects on Gene Flow

**DOI:** 10.3390/plants14131980

**Published:** 2025-06-28

**Authors:** Ning Hu, Dantong Wang, Qianhua Yuan, Yang Liu, Huizi Jiang, Xinwu Pei

**Affiliations:** 1 Yale-NUIST Center on Atmospheric Environment, State Key Laboratory on Climate System Prediction and Risk Management, Nanjing University of Information Science and Technology, Nanjing 210044, China; huning@nuist.edu.cn (N.H.); 202412080162@nuist.edu.cn (D.W.); 202312080016@nuist.edu.cn (Y.L.); 202412080146@nuist.edu.cn (H.J.); 2 Key Laboratory of Ecosystem Carbon Source and Sink-China Meteorological Administration, Nanjing University of Information Science and Technology, Nanjing 210044, China; 3 Collaborative Innovation Center on Forecast and Evaluation of Meteorological Disasters, Nanjing University of Information Science and Technology, Nanjing 210044, China; 4 College of Tropical Agriculture, Hainan University, Haikou 570228, China; qhyuan@163.com; 5 Biotechnology Research Institute, Chinese Academy of Agricultural Sciences, Beijing 100081, China

**Keywords:** gene flow rate, rice (*Oryza sativa* L.), pollen, quantitative competition, genetic competitiveness

## Abstract

The gene flow rate in rice (*Oryza sativa* L.) is a critical factor for establishing safe isolation distances between genetically modified (GM) and non-GM varieties and for ensuring varietal purity in rice breeding programs. This study refines existing gene flow models by disentangling two key components of rice pollen dynamics: quantitative pollen competition and genetic competitiveness. We define *B* as the proportion of GM pollen within mixed pollen, representing quantitative pollen competitiveness. The outcrossing parameter *C_b_* reflects the likelihood of successful fertilization and seed development by foreign pollen, while the hybrid compatibility parameter *C_p_* captures the relative fertilization success of GM versus non-GM pollen within the same pollen pool. Together, *C_b_* and *C_p_* characterize the genetic competitiveness of rice pollen. Our findings reveal a nonlinear relationship between *B* and the observed GM pollen rate *G*, which may exhibit either upward or downward curvature. A nonlinear model provides a significantly better fit to this relationship than a linear model, improving *R*^2^ by 4.1–21.4% and reducing *RMSE* by 9.9–47.8%. The parameters *C_b_* and *C_p_* play central roles in determining gene flow; higher values correspond to stronger GM pollen competitiveness, resulting in higher gene flow rates and greater dispersal distances. Specifically, *C_b_* sets the range of the *B*–*G* curve, while *C_p_* determines its curvature.

## 1. Introduction

Rice (*Oryza sativa* L.) is one of the primary staple crops worldwide. In 2023, the total rice cultivation area reached 168.36 million hectares, contributing about 19% of global grain production [1]. In recent years, substantial advances have been made in transgenic rice technology, and genetically modified rice varieties with enhanced traits, such as disease and insect resistance, have been successfully developed. Several of these varieties have completed safety evaluations and are nearing commercial deployment in China [2]. However, despite the potential benefits of transgenic technology, concerns remain regarding the ecological impacts and food safety risks associated with genetically modified rice [3,4,5]. Consequently, the issue of transgenic gene flow in rice has garnered growing attention, as it represents a critical factor in evaluating the ecological safety of genetically modified rice.

Transgene flow in rice refers to the transfer of target genes from genetically modified (GM) rice to nearby related species, leading to the incorporation of transgenic traits into these populations. This process can result in unintended trait modifications and the spread of target gene characteristics. The rate of transgene flow serves as a crucial reference for establishing appropriate isolation distances between GM and non-GM rice to ensure safe coexistence and maintain genetic integrity [6]. Transgene flow is typically quantified as the proportion of offspring in the receptor population that carries the target gene, representing the percentage of transgenic progeny among all descendant plants [7].

Field experiments are a key method for measuring transgene flow rates, and they are commonly conducted using circular or rectangular experimental designs [8,9,10,11,12]. In rectangular experimental designs, genetically modified (GM) rice and non-GM rice are planted either adjacently or in an intercropped pattern. Typically, GM rice is positioned upwind and non-GM rice downwind at varying distances to assess the extent of transgene flow. The primary advantage of the rectangular design lies in its efficiency—it conserves land and labor while enabling the estimation of maximum gene flow rates and their distribution over distance, even within relatively small plots. However, this design is highly dependent on wind direction. If the prevailing wind during the flowering period does not align with the intended orientation of the field layout, the experiment may fail to yield valid results. In contrast, the circular experimental design positions GM rice at the center, with non-GM rice planted in concentric rings surrounding it. This arrangement enables the collection of gene flow data across multiple directions and distances, effectively mitigating the risk of failure due to variable wind patterns. Field trial data indicate that the maximum gene flow rate between GM rice and conventionally cultivated rice when planted adjacently is generally less than 1% [8,9,10,11,12]. In contrast, the maximum gene flow rate from GM rice to male sterile lines varies significantly, ranging from 1.56% to 92.01%. The distance at which gene flow to male sterile lines falls below 1% ranges from 0 to 30 m [7]. Additionally, the maximum gene flow rate from GM rice to wild rice has been reported at 18% [7,13,14,15,16]. These findings offer valuable insights for establishing isolation distances for genetically modified rice in China. However, the scope of field trial data remains limited. Such data reflect gene flow dynamics under specific temporal, spatial, and genetic conditions—namely, particular environmental settings and specific donor–recipient combinations. As a result, they are not broadly applicable for predicting gene flow rates across diverse ecological contexts or with different combinations of GM and non-GM rice varieties.

Models can help address the limitations of field trial data and are valuable tools for evaluating regional gene flow risks associated with genetically modified rice [17]. Gene flow in rice encompasses not only the physical dispersal of pollen but also the biological processes of pollen germination, fertilization, and seed formation on the stigma. The number of GM pollen grains that land on the stigma plays a critical role in determining the probability of gene flow. Although only one pollen grain ultimately fertilizes the ovule, an increased number of pollen grains on the stigma enhances the likelihood of successful fertilization and seed set. Consequently, most gene flow models rely on the quantity of GM pollen deposition as a key predictor of gene flow potential [18]. However, pollen quantity alone does not fully determine gene flow rates. Genetic factors, such as the outcrossing rate of the recipient variety, the compatibility between the recipient’s stigma and the genetically modified donor pollen, and other genotype-specific traits, also play critical roles [17,19,20]. Empirical studies have demonstrated that even under similar environmental conditions and with comparable levels of GM pollen, gene flow rates can vary by up to 10^3^-fold between different rice varieties [7]. In rice, conspecific pollen typically exhibits a competitive advantage over foreign pollen [21]. To account for such variability, previous models have often introduced one or two constants to represent genetic competitiveness, assuming a linear relationship between GM pollen quantity and the gene flow rate [17,19,20]. However, our maize field trial data suggest that this relationship is frequently nonlinear and tends to follow a curvilinear pattern [22]. This raises important questions: How does gene flow in rice—an autogamous species—differ from that in cross-pollinated crops like maize? What role does pollen genetic competitiveness play in shaping gene flow dynamics in rice, and how does the gene flow rate relate to GM pollen density under varying genetic and ecological conditions?

To address these questions, this study investigates pollen competitiveness in rice using a dual-donor experimental design. This approach allows for the separation of genetic pollen competition from quantitative competitiveness, enabling the quantification of their individual contributions to gene flow rates. The ultimate goal is to improve the accuracy of gene flow modeling and to provide a robust data foundation for regional risk assessments of gene flow in genetically modified rice.

## 2. Results

### 2.1. Quantification of Pollen’s Quantitative and Genetic Competitiveness

Table 1 presents the results of the dual-donor experiment, which shows that even when the pollen ratio of GM and non-GM rice is the same, the resulting GM pollen rate (*G*) varies between different combinations. For example, when the B9:9311 ratio is 5:5, the resulting *G* is 62.35%, while for the B2: Xiushui 63 ratio at 5:5, *G* increases to 68.72%. This difference represents a relative strength index of genetic competitiveness for the GM rice pollen within the GM and non-GM pollen mixture. Under identical conditions, B2 and B9 demonstrate slightly stronger genetic competitiveness against Xiushui 63 and 9311, and the transgene flow rate from B2 to Xiushui 63 is a little higher than that from B9 to 9311. However, genetic competitiveness is relative and local, as it depends on one variety’s competitiveness against another in a given combination, and it may change when either component in the combination is altered. On the other hand, the process of fertilization and seed production is greatly influenced by temperature, humidity, etc., and the genetic competitiveness might vary under different environmental conditions, even if genetic contexts do not change.

Furthermore, as the quantitative pollen competitiveness (*B*) of GM pollen increases, the transgene flow rate (*G*) also increases; however, the relationship between *B* and *G* is nonlinear. When *B* is relatively small, *G* increases significantly as the GM pollen quantity rises. Yet, when *B* exceeds 50%, the growth rate of *G* gradually slows. Moreover, the relationship between *B* and *G* differs between the two combinations. *G* is significantly higher at low *B* values than in the lower-competitiveness combinations, where the GM rice has higher genetic competitiveness. Once *B* exceeds 0.8, the transgene flow rate differences between combinations with differing genetic competitiveness become minimal. This suggests that when GM and non-GM rice are planted adjacent to each other and GM pollen is dominant in quantity, genetic competitiveness differences have little impact on the transgene flow rate. However, at greater distances from GM rice, where the GM pollen quantity is lower, the effect of genetic competitiveness becomes more pronounced. Higher genetic competitiveness can substantially increase GM gene flow, leading to a greater risk of transgene spread.

To analyze the impact of hybrid compatibility on the transgene flow rate, we assumed an outcrossing rate of 90% for Bo A. The experimental results for different combinations were substituted into Equations (6) and (9). In the linear model, the hybrid compatibility parameter (*C_p_*) was 0.599 for B9 in Combination 1 and 0.603 for B2 in Combination 2. In the nonlinear model, *C_p_* was 0.693 for B9 and 0.764 for B2. Although the absolute values of *C_p_* differed between the two models, the overall trend remained consistent. In all cases, *C_p_* values were greater than 0.5, and the genetic competitiveness of B9 versus 9311 pollen was a little stronger than that of B2 versus Xiushui 63. This suggests that the transgene flow rate from B2 was also slightly higher than that from B9.

**Figure 1:**
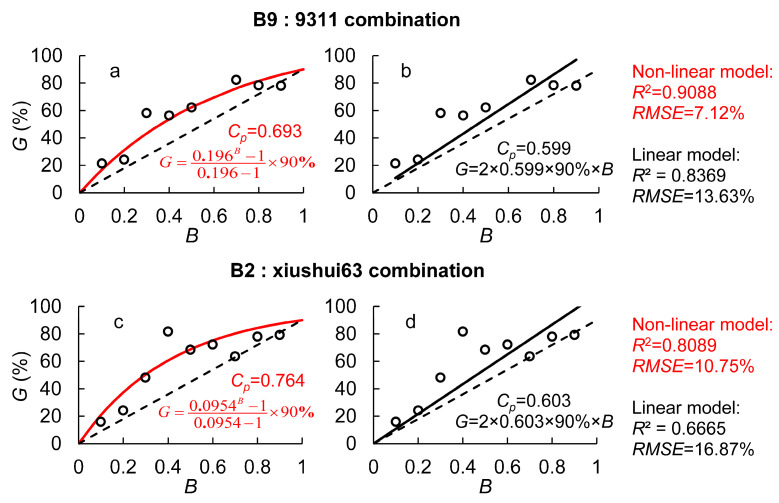
The transgene flow rate (*G*) under different proportions of GM pollen within mixed pollen (*B*) in the dual-donor experiment. (**a**,**b**) is the non-linear model and linear model for B9:9311 combination, respectively; (**c**,**d**) is the non-linear model and linear model for B2:xiushui63 combination, respectively; Red lines and words represent the results of the non-linear model and those in black represent the results from the linear model.

When comparing the performance of the two models, the nonlinear model demonstrated a notable improvement over the linear model. As shown in Figure 1, the coefficient of determination (*R*^2^) for the nonlinear model was 0.9088 (B9:9311) and 0.8089 (B2:Xiushui 63), representing an 8.6–21.4% increase compared to the linear model. Meanwhile, the root mean squared error (*RMSE*) was 7.12% (B9:9311) and 10.75% (B2:Xiushui 63), reflecting a 36.2–47.8% reduction relative to the linear model. Additionally, when GM pollen was dominant, both models exhibited varying degrees of overestimation of transgene flow rates, with the linear model showing more pronounced overestimation. In contrast, under low quantitative pollen competitiveness, the linear model consistently underestimated GM gene flow, particularly at greater distances. This led to an underestimation of the maximum gene flow distance, potentially resulting in an erroneous assessment of GM pollen risks.

### 2.2. Optimization of the Rice Gene Flow Model

To further analyze the relationship between quantitative pollen competitiveness, genetic competitiveness, and the gene flow rate, and to evaluate the predictive performance of linear and nonlinear models, this study utilized gene flow data from male sterile receptor lines in field experiments. First, the pollen dispersal model was applied to estimate the number of GM and non-GM rice pollen grains at different distances, from which the quantitative pollen competitiveness (*B*) was derived.

**Figure 2:**
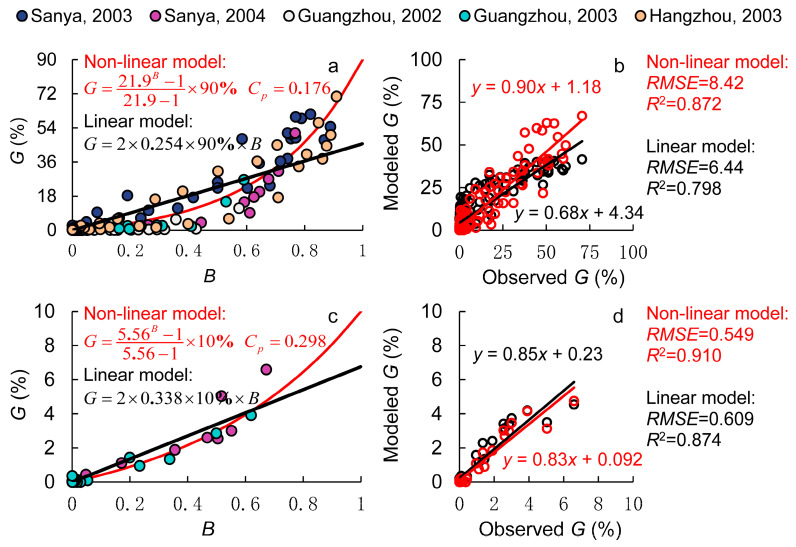
**Modeling pollen competitiveness in the gene flow field experiment.** (**a**,**b**) are the results of male sterile lines, where the dots with different colors represent the experiments in different sites and the lines in red and black represent the results of the non-linear and the linear model, respectively; (**c**,**d**) are the results of wild rice, where red lines and dots represent the results of the non-linear model and those in black represent the results from the linear model.

As shown in Figure 2a, a distinct nonlinear relationship was observed between quantitative pollen competitiveness (*B*) and the measured transgene flow rate (*G*). In the downwind area adjacent to GM rice, GM pollen dominated the mixed pollen, exhibiting the strongest quantitative pollen competitiveness, and the transgene flow rate was at its maximum. As the quantitative pollen competitiveness of GM pollen decreased, the transgene flow rate declined rapidly. However, this decline gradually slowed down, and once the GM pollen proportion fell below 50% (*B* < 0.5), the reduction in the transgene flow rate became much slower. This resulted in a downward-bending curve, contrasting with the upward-bending curve observed in the dual-donor experiment. This suggests that during short-distance dispersal, the transgene flow rate rapidly declines as *B* decreases, whereas in long-distance dispersal, the decline in the transgene flow rate is more gradual.

To quantify hybrid compatibility, an outcrossing rate of 90% was assumed, and all *B* and *G* values from the gene flow experiments were fitted using a nonlinear regression model. The results yielded *C_p_* = 0.176 for the nonlinear model and *C_p_* = 0.254 for the linear model, as shown in Figure 2. Compared with the results from the dual-donor experiment, *C_p_* values were significantly lower in both models, indicating that under the same pollen quantity conditions, L201 had a lower probability of fertilization and seed setting compared to other non-GM rice varieties. This may be related to subspecies differences between the donor and receptor rice varieties. L201 is an *Oryza sativa* cv. Japonica rice line, whereas Zhong 9A and Bo A are indica-type male sterile lines, which have lower hybrid compatibility than intra-subspecies crosses.

A comparison of the predictive performance of the nonlinear and linear models is presented in Figure 2b. The results show that the linear model significantly underestimated the transgene flow rate to male sterile receptor lines under high quantitative pollen competitiveness, while it overestimated the transgene flow rate under low competitiveness. In contrast, the nonlinear model demonstrated substantial improvements, reducing the *RMSE* between observed and predicted values by 23.8% while increasing *R*^2^ by 9.3%. However, despite these improvements, the nonlinear model still overestimated low gene flow rates and underestimated high gene flow rates. This may lead to an underestimation of the maximum gene flow rate and the gene flow distance. This discrepancy was primarily due to unexpectedly high gene flow rates observed in the 10–20 m downwind zone during the 2003 Sanya and Hangzhou experiments, which elevated the estimated *C_p_* values. The cause of these elevated gene flow rates remains unclear.

Next, we analyzed wild rice data from the gene flow experiment, specifically *Oryza rufipogon* (common wild rice, 2x, AA), the common ancestor of Asian cultivated rice. Because this wild rice species contains the AA genome, there are no interspecific reproductive barriers, allowing for natural hybridization with cultivated varieties. However, because the wild rice pollen is partially fertile compared to male sterile lines, transgene flow rates to wild rice were significantly lower under the same quantitative pollen competitiveness conditions, as shown in Figure 2c. Accordingly, we set *C_b_* to 10% for wild rice.

In addition, we compared the linear and nonlinear models. The results, shown in Figure 2d, demonstrate that the nonlinear model showed slight improvements in both *RMSE* and *R*^2^ over the linear model. In the 2004 Sanya experiment, the root mean squared error (*RMSE*) was kept within 0.5%. However, at short distances, both models significantly underestimated the transgene flow rates. For example, in the 2003 Guangzhou experiment, the maximum gene flow rate at 0 m was 18.0% (data excluded from the model), which was 1.8 times higher than the maximum transgene flow rate predicted by our model. This discrepancy arises because the outcrossing rate in the model was set at 10%, whereas actual outcrossing rates fluctuate. On the one hand, outcrossing rates vary by variety, influenced by factors like stigma exertion rate, pollen fertility, and flowering time synchronization. On the other hand, environmental conditions also play a role. High temperatures can reduce pollen viability, and meteorological variations can alter heading dynamics and flowering behavior, all of which impact fertilization and seed setting [23,24]. Therefore, outcrossing rates are not fixed and should be further refined in future research to enhance model accuracy.

### 2.3. Sensitivity Analysis of Rice Pollen Competitiveness

**Figure 3:**
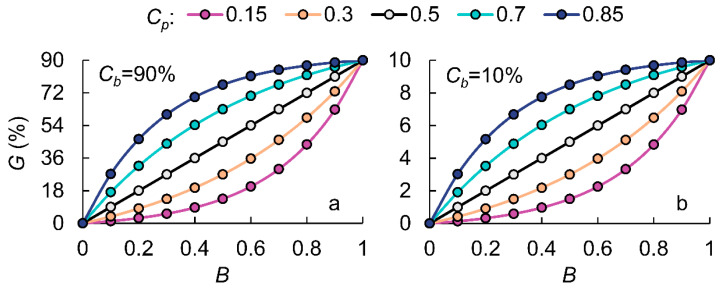
The modeled transgene flow rate (*G*) in response to the quantitative competitiveness of GM pollen (*B*) under different genetic competitiveness conditions (*C_p_*, *C_b_*).

To understand the impact of pollen competitiveness on transgene flow rates, this study conducted a sensitivity analysis by varying both genetic competitiveness (*C_p_*, *C_b_*) and quantitative pollen competitiveness (*B*) to assess changes in transgene flow rates (Figure 3). Firstly, changes in the outcrossing rate do not alter the relationship between *B* and *G*; rather, they cause a linear increase or decrease in the transgene flow rate across all values of *B*. Secondly, the hybrid compatibility parameter (*C_p_*) is a variable that reflects the likelihood of GM pollen successfully fertilizing receptor stigmas compared to non-GM pollen. When *C_p_* = 0.5, it indicates that GM pollen and non-GM pollen have equal genetic competitiveness. In this case, as GM pollen’s quantitative competitiveness (*B*) increases, the transgene flow rate (*G*) exhibits linear growth, forming a straight-line relationship between *B* and *G*.

However, in practice, very few experimental combinations have identical hybrid compatibility. When comparing four different hybrid compatibility levels (*C_p_* = 0.15, 0.3, 0.7, and 0.85), we found that when *C_p_* > 0.5, the relationship between *B* and *G* forms an upward-bending curve. The greater the *C_p_* value, the stronger the hybrid compatibility of GM rice, and the more pronounced the curvature of the curve. Conversely, when *C_p_* < 0.5, the relationship follows an opposite trend, indicating a downward-bending curve. This phenomenon suggests that when *B* is high, the impact of different *C_p_* values becomes more pronounced when *C_p_* < 0.5. Specifically, when GM pollen dominates the mixed pollen, gene flow rate differences between low-hybrid-compatibility varieties are more significant. In contrast, when non-GM pollen dominates, gene flow rate differences between high-hybrid-compatibility varieties become more pronounced.

To further analyze the impact of genetic competitiveness, data from the 2004 Sanya experiment were used to examine how pollen competitiveness influences gene flow by varying *C_p_* and *C_b_*. As shown in Table 2, the outcrossing rate (*C_b_*) is a critical factor affecting gene flow distance. When *C_b_* increased from 10% to 90%, the maximum threshold distances (MTD_1_% and MTD_0.1_%)—the distances at which transgene flow rates dropped below 1% and 0.1%, respectively—rose by 28% to 187%, with the effect being more pronounced for MTD_1_%. This is because transgene flow rates decline exponentially with increasing distance, and, at shorter distances, the rate of decline is steeper, making genetic competitiveness more influential. Additionally, the stronger the genetic competitiveness (*C_p_*) of GM pollen, the higher the transgene flow rate, resulting in an increase in threshold distances. This effect is especially noticeable for low-hybrid-compatibility varieties. When *C_p_* increased from 0.15 to 0.3, MTD_1_% increased by 35–51% and MTD_0.1_% increased by 49–76%. However, for high-hybrid-compatibility GM rice, MTD_1_% only increased by 8–22%, and MTD_0.1_% increased by 17–20%.

## 3. Discussion

To better characterize gene flow patterns among different genetic combinations, this study classified rice pollen competition into two distinct components: genetic competitiveness and quantitative competitiveness. These components were quantified to assess their respective effects on gene flow rates. The proportion of transgenic pollen in mixed pollen population *B* served as an indicator of quantitative competitiveness. The outcrossing parameter (*C_b_*) was introduced to represent the fertilization potential and subsequent seed production capacity of foreign pollen, while the hybrid compatibility parameter (*C_p_*) quantified the relative fertilization and seed production probability of transgenic pollen compared to non-transgenic pollen. Together, these parameters characterized the genetic competitiveness of rice pollen. Through dual-donor experiments and gene flow experiments, the relationships among *B*, *C_b_*, *C_p_*, and gene flow rates were established. The results demonstrate that while *C_b_* and *C_p_* values exhibit complexity and variability across different environmental and genetic backgrounds, a generalized nonlinear relationship between gene flow rate and quantitative competitiveness can be established. This relationship provides a predictive framework for assessing gene flow risks among various genetic combinations.

### 3.1. Quantitative Pollen Competitiveness in Rice

Quantitative pollen competition plays a crucial role in determining transgene flow rates. The greater the number of pollen grains deposited on the stigma, the higher the likelihood of fertilization and seed setting [25]. As the distance from GM rice increases, the concentration of airborne GM pollen decreases exponentially. This results in a lower proportion of GM pollen in the mixed pollen and, consequently, a reduction in the transgene flow rate. Field experiments on maize gene flow have shown that when receptor plants were detasseled at a 4:1 donor-to-receptor ratio, the 1% threshold distance increased from 35 m to 52 m [26]. Simulation results further revealed that reducing GM pollen by 50% led to a 69.3% decrease in the outcrossing rate and a 40% reduction in the 1% threshold distance. Conversely, reducing non-GM pollen by 50% increased the outcrossing rate by 70.9% and extended the 1% threshold distance by 20% (Hu Ning, PhD dissertation).

To better quantify the impact of variations in GM and non-GM pollen quantities on rice gene flow rates, we conducted a dual-donor experiment where a controlled ratio of GM and non-GM mixed pollen was manually applied to the stigmas of non-GM receptor plants. By measuring the transgene flow rate, we assessed the final fertilization ratio of GM and non-GM pollen. The study revealed a nonlinear relationship between quantitative pollen competitiveness (*B*) and the observed transgene flow rate (*G*), which formed either an upward- or downward-bending curve. This finding is consistent with results from maize studies [22], suggesting that a similar equation may describe gene flow in both crops, indicating a certain degree of universality. However, whether this equation can serve as a general model for other crops beyond rice and maize still requires further experimental validation.

Traditional gene flow models often describe the relationship between pollen competitiveness and gene flow rate using linear models [17], which can lead to model inaccuracies. This study compared the performance of linear and nonlinear models in predicting transgene flow rates and found that the nonlinear model showed significant improvements. In the dual-donor experiment, the nonlinear model increased *R*^2^ by 8.6–21.4% and reduced *RMSE* by 36.2–47.8%. In the gene flow field experiments, the nonlinear model reduced *RMSE* by 9.9–23.8% and increased *R*^2^ by 4.1–9.3%. More importantly, the nonlinear model corrected overestimation in cases where GM pollen was dominant and underestimation in cases where non-GM pollen was predominant—issues commonly encountered in the linear model.

### 3.2. Genetic Competitiveness of Rice Pollen

The number of pollen grains reaching the stigma often exceeds the number of ovules available for fertilization, resulting in pollen competition. The competition between self-pollinated and cross-pollinated pollen, along with the hybrid compatibility of GM pollen, plays a critical role in pollen-mediated gene flow [23,27,28]. This study advances previous research by introducing two genetic competitiveness parameters, *C_b_* and *C_p_*, into the rice gene flow model, while also considering pollen quantity competition. The outcrossing rate parameter (*C_b_*) reflects the probability of foreign pollen successfully fertilizing ovules, while the hybrid compatibility parameter (*C_p_*) quantifies the likelihood of GM pollen fertilizing ovules compared to non-GM pollen in the mixed pollen pool. Incorporating *C_b_* and *C_p_* not only enhanced the accuracy of transgene flow rate simulations but also provided a clearer explanation for the variation in gene flow rates across different rice varieties under identical experimental conditions.

Our study found that *C_p_* influences the curvature of the *B–G* curve (the relationship between pollen competitiveness, *B*, and the gene flow rate, *G*), while *C_b_* only affects the scale of the *B–G* curve without altering its shape. When *C_p_* = 0.5, GM rice and non-GM rice have equal hybrid compatibility, resulting in a linear 1:1 relationship between the pollen quantity ratio and the gene flow rate. A larger curvature indicates a greater difference in hybrid compatibility between GM and non-GM rice. When *C_p_* > 0.5, GM pollen exhibits higher hybrid compatibility than non-GM pollen, causing the curve to bend upward. A higher *C_p_* value indicates stronger hybrid compatibility of GM rice. Conversely, when *C_p_* < 0.5, GM pollen has lower competitiveness than non-GM pollen, causing the curve to bend downward, with a lower *C_p_* value reflecting weaker hybrid compatibility of GM rice.

Genetic competitiveness plays a crucial role in determining gene flow. The higher the values of *C_b_* and *C_p_*, the greater the GM pollen rate. As *C_b_* increases from 10% to 90%, the threshold distances, MTD_1_% and MTD_0.1_% (where GM pollen rates are ≤1% and ≤0.1%, respectively), increase by 28% to 187%, with a more significant effect on MTD_1_%. When *C_p_* rises from 0.15 to 0.3, MTD_1_% increases by 35% to 51%, while MTD_0.1_% increases by 49% to 76%. However, for GM rice with high outcrossing compatibility, the increases are smaller, as MTD_1_% rises by only 8% to 22% and MTD_0.1_% increases by just 17% to 20%.

Hybrid compatibility varies across different experimental material combinations. For GM rice varieties B9 and B2, the hybrid compatibility parameters are *C_p_* = 0.693 and 0.764, respectively, indicating that these varieties have slightly higher hybrid compatibility than non-GM rice varieties 9311 and Xiushui 63. As a result, B9 and B2 exhibit a higher probability of successful fertilization and seed setting, leading to increased gene flow rates and longer threshold distances. In contrast, for GM rice variety L201, *C_p_* = 0.176 and 0.298. Because *C_p_* < 0.5, L201 has weaker hybrid compatibility compared to other non-GM varieties in the experiment, resulting in a lower probability of fertilization, reduced gene flow rates, and shorter threshold distances.

The wide compatibility trait of a variety significantly influences gene flow between subspecies. Research has demonstrated that there is post-zygotic reproductive isolation between the indica and japonica subspecies of Asian cultivated rice, leading to poor hybrid compatibility. The F1 hybrids between subspecies typically exhibit high pollen sterility, with seed setting rates ranging from only 10% to 30%. In contrast, F1 hybrids between different ecotypes within the same subspecies tend to have much higher seed setting rates, usually exceeding 70%, indicating better compatibility [29]. This explains why, in the Sanya and Guangzhou experiments, the gene flow rate from the japonica rice variety L201 to the indica sterile line Bo A was relatively low. However, some varieties carry wide compatibility genes that allow F1 hybrids between indica and japonica to achieve higher seed setting rates. In recent years, a large number of wide compatibility varieties have been developed in China. Compared to varieties lacking these wide compatibility genes, such varieties exhibit higher genetic competitiveness, thereby posing a greater risk for gene flow.

### 3.3. Self-Pollination and Cross-Pollination

In nature, both cross-pollinating and self-pollinating plants exist, and maize (corn) is a prominent example of a monoecious, cross-pollinating species with a naturally high outcrossing rate exceeding 95%. In maize, the male flowers, known as tassels, develop at the top of the plant, while the female flowers, known as ears, grow on the upper stem nodes. When the male flowers release pollen, it is carried by the wind to the silks of female flowers, where GM and non-GM pollen compete for fertilization. Pollen with greater competitiveness has a higher chance of fertilizing the female flowers, leading to the potential spread of transgenes. To model the gene flow of GM pollen in maize, we developed a GM gene flow model that incorporates factors like pollen dispersal, genetic competitiveness, and the proportion of GM pollen within the total pollen mix. This model is designed to quantify the impact of pollen competitiveness on the transgene flow rate, helping to predict the spread of GM traits in maize populations [22]:

(1) $$G=\frac{{\left[(1-C_{p})/C_{p}\right]}^{2B}-1}{{\left[(1-C_{p})/C_{p}\right]}^{2}-1}\times 100\%$$

This model does not explicitly consider the effects of self-pollination and cross-pollination in maize. Instead, it determines the probability of fertilization and seed setting solely based on the proportion of pollen that reaches the silks. In previous work, we introduced a new variable, the genetic competitiveness parameter, into the maize gene flow model. This parameter describes the preference when GM and non-GM pollen both land on the same silk of a non-transgenic maize flower, known as hybrid compatibility [22].

Rice, in contrast to maize, is a hermaphroditic, self-pollinating plant, where self-pollen typically holds a significant fertilization advantage over foreign pollen. The natural outcrossing rate in rice is usually quite low, around 0.2% to 0.3%, although it can exceed 1% when different varieties are planted adjacent to each other. Additionally, male sterile lines, which are widely used in rice production, can exhibit outcrossing rates higher than 70% [30]. Given this, this study assumes that a fixed proportion of ovules undergoes self-fertilization before any outcrossed pollen arrives. The remaining ovules are then available for fertilization by foreign pollen [19]. To account for this dynamic, the rice gene flow model incorporates the outcrossing rate parameter (*C_b_*) into Equation (1). In this model, foreign GM pollen competes not only with non-GM foreign pollen but also with the self-pollen produced by the receptor plant. The likelihood of successful fertilization by GM pollen depends on its compatibility with the stigma, which is represented by the hybrid compatibility parameter (*C_p_*) in the model. Both *C_p_* and *C_b_* serve as indicators of the genetic competitiveness of rice pollen and are crucial factors that influence the transgene flow rate.

## 4. Material and Methods

In this study, pollen competitiveness is dissected into two components: quantitative pollen competitiveness and genetic competitiveness. Quantitative pollen competitiveness refers to the variation in gene flow rates resulting from differences in pollen quantity when pollen from different sources shares the same genetic background. In contrast, genetic competitiveness refers to the variation in gene flow rates due to differences in genetic background when the donor and other pollen types are present in equal quantities. To quantify quantitative pollen competitiveness, a one-dimensional inverse Gaussian model is employed to simulate the spatial distribution of rice pollen. Additionally, a genetic competitiveness parameter is introduced to enhance the accuracy of the rice gene flow model.

### 4.1. Data Sources

The data used in this study were obtained from several studies. Specifically, the dual-donor experiment was conducted in Sanya, Hainan (18°14′ N, 109°31′ E, 2018), from 2016 to 2017, which was detailed in Zhao et al. [31]. The gene flow experiments were conducted in Guangzhou, Guangdong (23°10′ N, 113°20′ E, 2002–2003), Sanya, Hainan (18°14′ N, 109°31′ E, 2003–2004), and Hangzhou, Zhejiang (30°14′ N, 120°10′ E, 2003), from 2002 to 2005, detailed in the references of Pei et al. [32]. In these experiments, the bar gene was used as the marker to detect the gene flow rate.

#### 4.1.1. Dual-Donor Experiment

**Figure 4:**
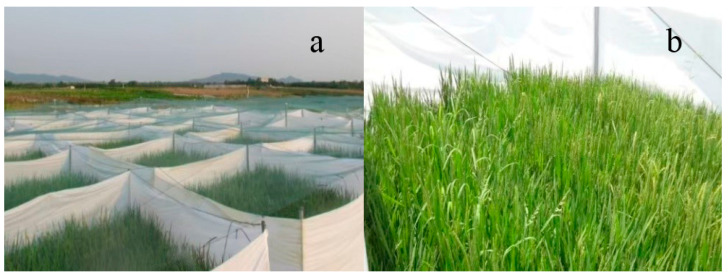
Photographic illustration of the dual-donor experiment. (**a**) is a panoramic image and (**b**) is the partial view of a treatment.

The dual-donor experiment (Figure 4) was designed to separate quantitative pollen competitiveness from genetic competitiveness by comparing gene flow rates under specific pollen ratio conditions. This approach allows for an independent analysis of the effects of quantitative pollen competition and genetic competitiveness on gene flow rates.

In this experiment, two donor combinations were used. In Combination 1, the donor materials consisted of herbicide-resistant indica rice B9 carrying the bar gene and conventional indica rice 9311. In Combination 2, the donor materials included herbicide-resistant japonica rice B2 carrying the bar gene and conventional japonica rice Xiushui 63. The receptor rice in both cases was the male sterile line Bo A. The dual-donor rice was planted adjacent to the receptor rice across 11 different pollen ratio treatments, specifically, 0:10, 1:9, 2:8, 3:7, 4:6, 5:5, 6:4, 7:3, 8:2, 9:1, and 10:0. To prevent cross-contamination between different treatments, fabric barriers were used for flowering phase isolation.

Because B9 and B2 were derived by introducing the bar gene into 9311 and Xiushui 63, respectively, the two donors exhibit similar agronomic traits. As a result, it can be assumed that their effective tiller number, spikelet number per panicle, and pollen quantity per spikelet are nearly identical. Therefore, the ratio of GM to non-GM pollen deposited on the receptor stigma closely mirrors the designed planting ratios of 0:10, 1:9, 2:8, 3:7, 4:6, 5:5, 6:4, 7:3, 8:2, 9:1, and 10:0.

#### 4.1.2. Gene Flow Experiments

**Figure 5:**
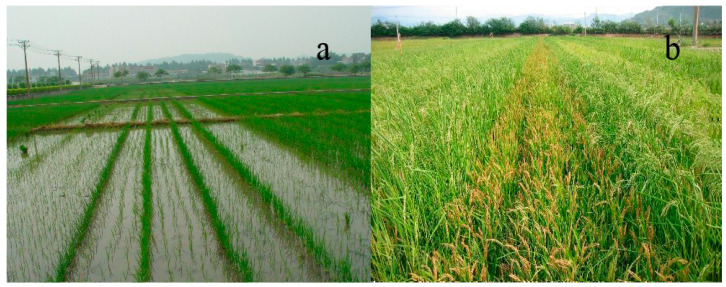
Photographic illustration of the gene flow experiment. (**a**) is the image after transplanting and (**b**) was taken during the filling stage.

This study introduces the concept of genetic competitiveness and quantifies the relationship between quantitative pollen competitiveness and the transgene flow rate to refine the rice gene flow model. To compare the performance of linear and nonlinear models, data from a rectangular gene flow experiment, as shown in Figure 5 are utilized.

The experiments were conducted in the field more than 1 km away from other rice plants or the flowering stages of experimental materials were more than 30 days apart within a 200 m radius. The field was aligned with the prevailing wind direction during the flowering stage, with 80–340 m in length and 70–80 m in width. In the upwind direction, within a range of 8–40 m, pollen donor rice and receptor rice were planted adjacently in a fixed row ratio, referred to as the 0 m zone. Starting from the downwind edge of the 0 m zone, receptor rice varieties were planted in parallel at specific intervals: every 1 m within the 1–5 m range, every 2.5 m within the 6–10 m range, every 10 m within the 10–50 m range, every 25 m within the 50–150 m range, and every 50 m beyond 150 m. Pollen competition sources were planted within each interval to ensure a uniform rice field surface.

In this experiment, the pollen donor was L201, a japonica rice variety carrying the bar gene for Basta herbicide resistance. The receptor plants included three types of male sterile lines, along with common wild rice, conventional rice, and hybrid rice. A total of five conventional and hybrid rice varieties were used: Yuezha 922, Yuezha 889, Peizha 77, Texianzhan 25, and Wuyujing. Additionally, two three-line male sterile lines (Zhong 9A and Bo A) and one common wild rice variety (planted only in Sanya and Guangzhou) were included. Among these receptor materials, conventional and hybrid rice typically exhibit outcrossing rates of less than 1%, making them representative of low outcrossing rates. Male sterile lines, theoretically, do not produce viable pollen. In the absence of non-GM pollen competition, the gene flow rate to male sterile lines approaches 100%, making them representative of high outcrossing rates. Wild rice falls between these two categories, as some plants in the common wild rice population exhibit partial or complete sterility, and the high stigma exertion rate of wild rice increases its receptivity to foreign pollen [7].

#### 4.1.3. Detection of Gene Flow Rate

**Figure 6:**
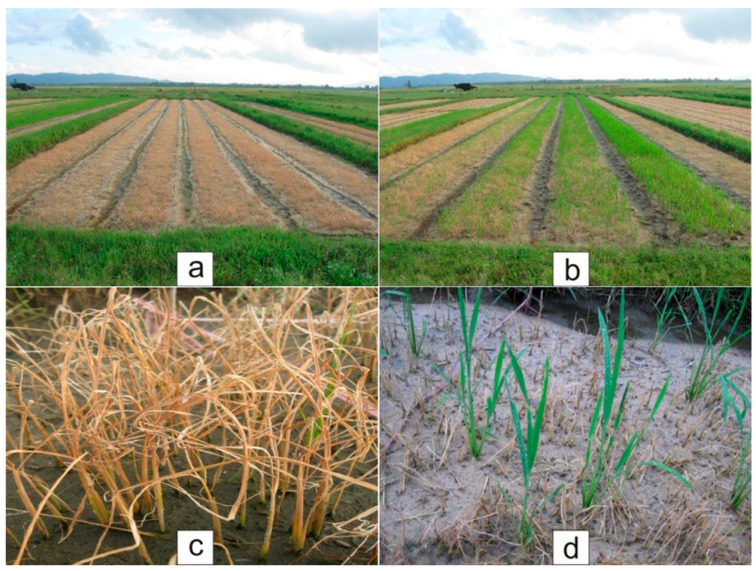
Photographic illustration of the detection of Basta herbicide resistance. (**a**,**b**) are panoramic images. (**c**) shows that seedlings not containing the *bar* gene turned yellow and died after being sprayed with Basta herbicide. (**d**) shows that the seedlings carrying the *bar* gene remained green after being sprayed with Basta herbicide.

After maturation, the seeds on receptors were collected from each experiment and sown in the paddy fields elsewhere. When the seedlings grew their third leaf, the Basta herbicide (BAYER Group, Leverkusen, Germany) was sprayed three times, with each spraying interval lasting 7 days. Seedlings without the bar gene turned yellow and died after being sprayed with Basta, while seedlings carrying the bar gene remained green, as shown in Figure 6, indicating that they were derived from GM gene flow. One week after spraying, the number of Basta-resistant seedlings and the total seedlings were recorded. The gene flow rate was calculated as the ratio of Basta-resistant (Basta^R^) surviving plants to the total number of seedlings following herbicide application.

### 4.2. Rice Gene Flow Model

#### 4.2.1. Simulating Pollen Diffusion

The inverse Gaussian distribution is a widely used statistical model that has been applied to characterize the dispersal patterns of wind-dispersed plant seeds and pollen [33]. A key distinction between the inverse Gaussian model and other negative exponential models lies in its ability to capture near-source dispersal dynamics. While traditional negative exponential models assume a monotonous decline in pollen concentration with distance from the source, the inverse Gaussian model allows for an initial increase in pollen concentration followed by a decline along the wind’s direction. This feature more accurately reflects the spatial patterns observed in our field experiments, making it a more suitable choice for modeling rice pollen dispersal.

(2) $$D(x)=\sqrt{\frac{\lambda }{2\pi x^{3}}}\exp[-\frac{\lambda {\left(x-\mu \right)}^{2}}{2\mu^{2}x}]$$

In the equation, *x* represents the dispersal distance from the pollen donor to the receptor rice (in meters), and *D*(*x*) denotes the pollen density at location *x* (grains·m^−2^). The parameter *λ* is the scale parameter, while *μ* is the location parameter, which can be expressed as

(3) $$\lambda =\frac{hU_{H}}{2\kappa \sigma_{w}}$$

(4) $$\mu =\frac{hU_{H}}{V_{t}}$$

In the equation, *h* (in meters) represents the relative release height of the pollen, defined as the difference between the pollen release height and the stigma height of the receptor rice. For this study, *h* is set to 0.2 m. *κ* is the Kármán constant at 0.4. *V_t_* (m·s^−1^) represents the pollen deposition velocity, set as 0.08 m·s^−1^ [17]. $\sigma_{w}$ denotes the standard deviation of the vertical wind speed, calculated as $1.1\times u_{*}$, which can be estimated from the wind speed at a height of 10 m measured at a nearby meteorological station, the zero-plane displacement (*d*, set as 0.63*H*), and the roughness length ($z_{0}$, set as 0.13*H*) from Equation (5), and then $U_{H}$ (m·s^−1^), the mean wind speed at canopy height, can also be obtained from this equation:

(5) $$U=\frac{u_{*}}{\kappa }\ln(\frac{z-d}{z_{0}})$$

#### 4.2.2. Simulating Gene Flow

The transgene flow rate is influenced by two primary factors, quantitative pollen competitiveness and genetic competitiveness. To describe the relationship between these factors, a linear equation is employed, expressed as

(6) $$G=2C_{p}\times C_{b}\times B\times 100\%$$

In the equation, *G* represents the transgenic gene flow rate (%), while *C_p_* and *C_b_* reflect the genetic competitiveness of transgenic rice. *C_p_* denotes the hybrid compatibility of transgenic rice relative to non-transgenic rice, with values ranging from 0 to 1. A value of *C_p_* = 0.5 indicates equal hybrid compatibility between the two; if *C_p_* > 0.5, transgenic rice exhibits stronger hybrid compatibility; if *C_p_* < 0.5, non-transgenic rice has stronger hybrid compatibility. *C_b_* represents the outcrossing rate (%) of the receptor rice. For male sterile lines, the theoretical outcrossing rate is 100% due to the absence of viable pollen; however, in practice, some viable pollen is still produced, so *C_b_* is set to 90%. For wild rice, *C_b_* is set to 10% [7]. *B* represents the quantitative pollen competitiveness of GM pollen, indicating its proportion within the mixed pollen, and it is expressed as $B=P_{Tr}/(P_{Tr}+P_{Cr})$, where *P* denotes the number of viable pollen grains and the subscripts *Tr* and *Cr* represent transgenic rice and non-transgenic rice, respectively (grains). *P* is given by

(7) P=D⋅Λ

In Equation (7), *D* represents the number of pollen grains deposited on the stigma of the receptor rice (in grains), while *Λ* denotes pollen viability (%). In rice, pollen typically has a short lifespan, losing viability within 3–6 min after dispersal from the anther, with only a small proportion of grains surviving for 15–30 min. In contrast, wild rice pollen has a longer lifespan, remaining viable for up to 9 min [34]. In this study, logistic regression was used to model rice pollen viability:

(8) $$\Lambda =\Lambda_{\max}/[1+e^{a(t-b)}]$$

In Equation (8), *t* represents the time elapsed after pollen is released from the anther (in minutes), and *Λ_max_* denotes the maximum pollen viability, set at 98.5%. The parameters *a* and *b* are empirical constants; for cultivated rice, *a* is set to 0.904 and *b* to 4.834, while for wild rice, *a* is set to 0.452 and *b* to 9.668 [32].

When *B* = 0, the mixed pollen consists solely of non-GM pollen, resulting in *G* = 0%. When *B* = 0.5, the quantities of GM and non-GM pollen in the mixture are equal, and the gene flow rate is given by *G* = *C_p_* × *C_b_* × 100%. When *B* = 1, the mixed pollen consists entirely of GM pollen, leading to the maximum gene flow rate. According to Equation (6), *G* = 2*C_p_* × *C_b_* × 100%. However, theoretically, *G* should equal the outcrossing rate, i.e., *G* = *C_b_* × 100%. To account for these boundary conditions, Equation (6) is optimized into the following nonlinear equation:

(9) $$G=\frac{{\left[(1-C_{p})/C_{p}\right]}^{2B}-1}{{\left[(1-C_{p})/C_{p}\right]}^{2}-1}\times C_{b}\times 100\%$$

#### 4.2.3. Model Validation

To evaluate the performance of the gene flow model and compare the effectiveness of the linear and nonlinear equations, this study employs two common statistical metrics: the root mean squared error (*RMSE*) and the coefficient of determination (*R*^2^). These metrics are used to assess the agreement between the simulated and observed values. The calculation methods for *RMSE* and *R*^2^ are as follows:

(10) $$RMSE=\sqrt{\frac{\sum_{i=1}^{n}{\left(SIM_{i}-OBS_{i}\right)}^{2}}{n}}$$

(11) $$R^{2}=\frac{\sum_{i=1}^{n}{\left(SIM_{i}-\bar{SIM}\right)}^{2}}{\sum_{i=1}^{n}{\left(OBS_{i}-\bar{OBS}\right)}^{2}}$$

In the equation, *n* represents the sample size, *OBS_i_* denotes the observed value, and $\bar{OBS}$ is the mean of the observed values. *SIM_i_* represents the simulated value, and $\bar{SIM}$ is the mean of the simulated values.

## Figures and Tables

**Table 1:** Transgene flow rate (*G*) under different levels of quantitative pollen competitiveness (*B*) in the dual-donor experiment.

*B*	*G* (%)	
	B9: 9311	B2: Xiushui63
0.1	21.23	15.92
0.2	24.20	24.16
0.3	58.01	48.13
0.4	56.26	81.66
0.5	62.12	68.42
0.6	-	72.18
0.7	82.23	63.61
0.8	78.36	77.96
0.9	77.94	79.18

**Table 2:** Threshold distances (MTD_1_% and MTD_0.1_%) for GM gene flow in rice under different *C_p_* (0.15, 0.30, 0.50, 0.70, 0.85) and *C_b_* (10%, 90%) conditions.

	*C_p_*	0.15	0.3	0.5	0.7	0.85
MTD_1%_	*C_b_* = 90%	46.8	70.5	89.3	99.6	121.3
	*C_b_* = 10%	16.3	28.7	43.6	59.3	69.5
MTD_0.1%_	*C_b_* = 90%	91.8	123.7	142.4	148.4	160.3
	*C_b_* = 10%	48.4	71.8	91.3	103.5	124.6

Note: MTD_1_% and MTD_0.1_% represent the distances at which the transgene flow rate is lower than or equal to 1% and 0.1%, respectively.

## Data Availability

The original contributions presented in this study are included in the article. Further inquiries can be directed to the corresponding author.
